# Regulation of Expression of *CEBP* Genes by Variably Expressed Vitamin D Receptor and Retinoic Acid Receptor α in Human Acute Myeloid Leukemia Cell Lines

**DOI:** 10.3390/ijms19071918

**Published:** 2018-06-29

**Authors:** Aleksandra Marchwicka, Ewa Marcinkowska

**Affiliations:** Department of Biotechnology, University of Wroclaw, Joliot-Curie 14a, 50-383 Wroclaw, Poland; alexandramarchwicka@interia.pl

**Keywords:** nuclear receptors, CCAAT-enhancer-binding proteins, *CEBP* genes, vitamin D receptor, retinoic acid receptor α, differentiation, acute myeloid leukemia

## Abstract

All-*trans*-retinoic acid (ATRA) and 1α,25-dihydroxyvitamin D (1,25D) are potent inducers of differentiation of myeloid leukemia cells. During myeloid differentiation specific transcription factors are expressed at crucial developmental stages. However, precise mechanism controlling the diversification of myeloid progenitors is largely unknown, CCAAT/enhancer-binding protein (C/EBP) transcription factors have been characterized as key regulators of the development and function of the myeloid system. Past data point at functional redundancy among C/EBP family members during myeloid differentiation. In this study, we show that in acute myeloid leukemia (AML) cells, high expression of vitamin D receptor gene (*VDR*) is needed for strong and sustained upregulation of *CEBPB* gene, while the moderate expression of *VDR* is sufficient for upregulation of *CEBPD* in response to 1,25D. The high expression level of the gene encoding for retinoic acid receptor α (*RARA*) allows for high and sustained expression of *CEBPB*, which becomes decreased along with a decrease of *RARA* expression. Expression of *CEBPB* induced by ATRA is accompanied by upregulated expression of *CEBPE* with similar kinetics. Our results suggest that *CEBPB* is the major VDR and RARA-responsive gene among the *CEBP* family, necessary for expression of genes connected with myeloid functions.

## 1. Introduction

CCAAT/enhancer-binding proteins (C/EBPs) are transcription factors that activate the expression of target genes through interaction with response elements within their promoters [1]. There are six members of C/EBP family, and they regulate differentiation process in various tissues [2]. The first transcription factor in this family, C/EBPα, was isolated from the rat liver and it appeared to be important for adipocyte differentiation [3]. C/EBPs are modular proteins consisting of an activation domain, a DNA binding domain, and a leucine-rich dimerization domain that is responsible for forming dimers with other members of the family [4]. In order to activate transcription, the C/EBP dimers bind to the consensus sequence 5′-TT/GNNGNAAT/G-3′ in promoter regions of target genes. For three out of six genes encoding C/EBP family members, alternative protein products are translated, due to a leaky ribosomal scanning mechanism. Some of them lack the N-terminal activation domains and exert inhibitory functions, presumably by a dominant negative mechanism [5].

Hematopoiesis is a process in which all blood elements are formed from multipotential hematopoietic stem cells (HSCs). In the process of hematopoiesis, the HSCs and their progeny interact with the bone marrow stromal cells and they are stimulated by the numerous growth factors that are secreted in the bone marrow environment. The eventual cell fate during hematopoiesis is governed by spatiotemporal fluctuations in transcription factor concentrations, which either cooperate or compete in driving target gene expression [6]. Some members of C/EBP family of transcription factors are important at certain steps of hematopoiesis [7]. C/EBPα appears in differentiating cells at the stage of early progenitors with lymphoid and myeloid potential and then reappears only in the cells that are differentiating into granulocytes [8]. C/EBPα-deficient mice show disturbances in monocyte and neutrophil development [9,10]. High level of C/EBPβ leads to monocyte and macrophage development [11,12], while high level of C/EBPε leads to neutrophil differentiation [13]. The role of C/EBPδ in blood cells development is less defined, since *CEBPD*−/− mice did not reveal any blood disturbances [14]. It has been documented that C/EBPδ regulates expression of genes important for granulocyte function [15].

However, the most important factors that drive blood cells development are cytokines [16], some ligands for nuclear receptors can also modulate cell fate during hematopoiesis [7]. The best described in this respect are ligands for retinoic acid receptors (RARs). Active metabolites of vitamin A are natural ligands for RARs. A dominating retinoic acid (RA) metabolite is all-*trans*-RA (ATRA), which binds with high affinity to all RARs (α, β, and γ) [17]. During embryogenesis, ATRA causes the appearance of hematopoietic progenitors from the hemogenic endothelium [18], while in adults, it is important for the differentiation of granulocytes, as well as B and T lymphocytes [19]. This activity of ATRA has been used in clinics. The most clinically significant application of ATRA is to treat a rare subtype of an acute myeloid leukemia (AML), called acute promyelocytic leukemia (APL). At the first description this subtype was considered the most difficult to treat [20], while it is now considered as highly curable using the combination of ATRA and anthracycline-based chemotherapy [21]. Another ligand for the nuclear receptor which influences hematopoiesis is an active metabolite of vitamin D. The correct physiological concentrations of 1,25-dihydroxyvitamin D (1,25D), which is a natural ligand for vitamin D receptor (VDR), are necessary to induce markers of monocytic differentiation in HSCs [22]. The expression of the *VDR* gene is higher at the early steps of hematopoiesis than at later stages and in mature blood cells [23]. However, both these ligands do not seem indispensable for blood cells development since RARα-deficient and VDR-deficient mice show no defects in hematopoiesis [24,25]. The possibility that these nuclear receptors can, in some aspects, functionally compensate each other should be considered.

It has been documented in the past that members of C/EBP family of transcription factors can be upregulated in blood cells by an exposure to RA, 1,25D, or to their active analogs. For example, the expression of C/EBPε mRNA and protein increases in AML cells exposed to 9-*cis*–RA or 1,25D analog (KH1060) [26]. The gene encoding C/EBPβ has been shown to be a target for VDR regulation [27] and all isoforms of this transcription factor are increased in AML cells exposed to 1,25D or to analogs of 1,25D [11,28]. This gene is also strongly upregulated in AML cells that were exposed to ATRA [29]. 1,25D induces a transient increase of C/EBPα [11], which also participates in the ATRA-induced differentiation of AML cells [30].

In this study, we addressed a question of whether the lack of one of the nuclear receptors mentioned above could be compensated by the other in terms of *CEBP* activation. Therefore, we used four cell lines in our study, with different expression of retinoic acid receptor α (*RARA*) or *VDR*. In HL60 cells, *VDR* expression is on a high level and *RARA* is moderate [31]. For the purpose of this study, we silenced the expression of *VDR* in HL60 cells using shRNA. In contrast to HL60 cells, KG1 cell express high levels of *RARA*, but low of *VDR* [31]. The effects of *RARA* silencing were studied using a sub-line KG1-RARα(−).

## 2. Results

### 2.1. Activation of Expression of CEBP Transcription Factor’s Genes in AML Cells with High Level of VDR and Low Level of RARα

**Figure 1:**
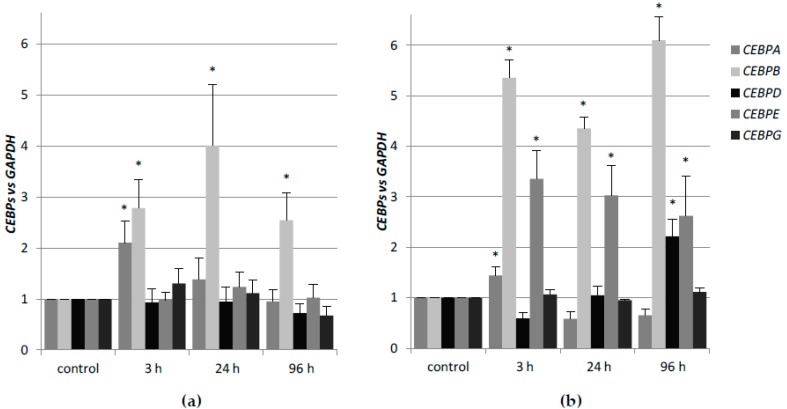
Regulation of *CEBP* genes in HL60 exposed to 1,25D or all-*trans*-retinoic acid (ATRA). HL60 cells were exposed to 10 nM 1,25D (**a**) or 1 µM ATRA (**b**) and after desired time the expression of *CEBP* genes was measured by Real-time PCR. The bars represent mean values (±standard error of the mean (SEM)) of the fold changes in mRNA levels relative to glyceraldehyde 3-phosphate dehydrogenase (*GAPDH*) mRNA levels. Expressions in control cells were treated as calibrators. Values significantly different from those obtained from respective controls cells are marked with an asterisk (* *p* < 0.05).

In previous studies, we have shown that different AML cell lines have variable sensitivity to 1,25D− and ATRA-induced differentiation [32]. HL60 cell line responded to 1,25D with robust monocytic differentiation and to ATRA with moderate granulocytic differentiation. That corresponded to high basal level of expression of *VDR* and low basal level of expression of *RARA* [31]. In view of demonstrated regulation of differentiation of myeloid leukemia cells by these two compounds, it was of interest to determine the expression profiles of *CEBP* genes in response to 1,25D and ATRA in HL60 cells. Therefore, the expression of *CEBPA*, *CEBPB*, *CEBPD*, *CEBPE*, and *CEBPG* in HL60 cells that were exposed to 1 μM ATRA or to 10 nM 1,25D for different time periods was tested. As depicted in Figure 1a, transient upregulation of *CEBPA* was detected in HL60 cells stimulated with 1,25D, followed by fast decline. This was in concordance with an earlier observed transient upregulation of C/EBPα protein in HL60 cells after exposure to 1,25D [11]. The increase in expression of *CEBPB* was more sustained, with a peak at 24 h and more gradual decline. As presented before, protein level of C/EBPβ follows this sustained expression pattern and it peaks between two and three days of exposure to 1,25D [11]. *CEBPD*, *CEBPE*, and *CEBPG* were not upregulated in response to 1,25D exposure of HL60 cells. As presented in Figure 1b in HL60 cells that were exposed to ATRA, *CEBPA* was upregulated weakly and transiently. Expression of *CEBPB* and *CEBPE* was stimulated by *ATRA* stronger and in a sustained manner. Modest upregulation of *CEBPD* was observed at 96 h from exposure to ATRA. Again, no stimulation of *CEBPG* was observed. Values of mRNA expression obtained using comparative quantification algorithm are presented in Table A1.

### 2.2. Activation of Expression of CEBP Transcription Factor’s Genes in AML Cells with Low Level of VDR and High Level of RARα

**Figure 2:**
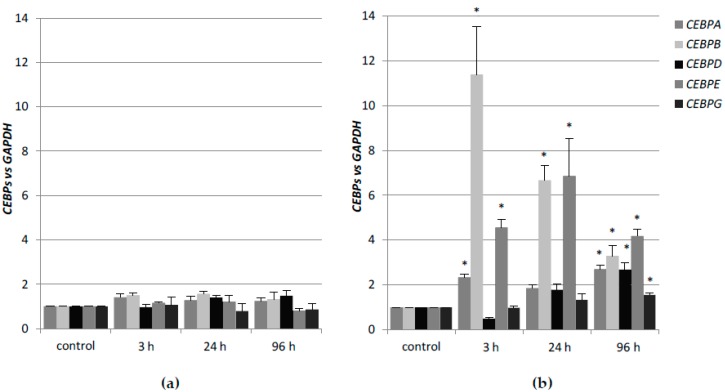
Regulation of *CEBP* genes in KG1 exposed to 1,25D (**a**) or to ATRA (**b**). KG1 cells were exposed to 10 nM 1,25D or 1 µM ATRA and after desired time the expression of *CEBP* genes was measured by Real-time PCR. The bars represent mean values of the fold changes (±SEM) in mRNA levels relative to *GAPDH* mRNA levels. Expressions in control cells were treated as calibrators. Values significantly different from those that were obtained from respective controls cells are marked with asterisk (* *p* < 0.05).

In contrast to HL60 cells, KG1 cells are not responsive to 1,25D and they have a low level of VDR protein, whilst being susceptible to ATRA-driven granulocytic differentiation [33]. This corresponds with the high basal level of expression of *RARA* gene and high constitutive content of RARα protein [31]. In the next series of experiments, KG1 cells were exposed to 1 μM ATRA or to 10 nM 1,25D for different time periods. In KG1 cells, the transcript levels of *CEBP* genes remained unchanged after exposure to 1,25D (Figure 2a). In contrast, significant changes in expression of *CEBP* genes after exposure of KG1 cells to ATRA were observed. Modest upregulation of *CEBPA* was detected at 3 h and 96 h from exposure. *CEBPB* was the most responsive to ATRA out of the genes studied, the expression upregulation was fast and long-lasting. The second ATRA-responsive gene was *CEBPE*, where the expression peaked at 24 h. The expression of *CEBPD* and *CEBPG* was modest with a peak at 96 h (Figure 2b). Values of mRNA expression that were obtained using comparative quantification algorithm are presented in Table A2.

### 2.3. Effects of Silencing High RARA on Expression of CEBP Transcription Factor’s Genes in KG1 Cells

**Figure 3:**
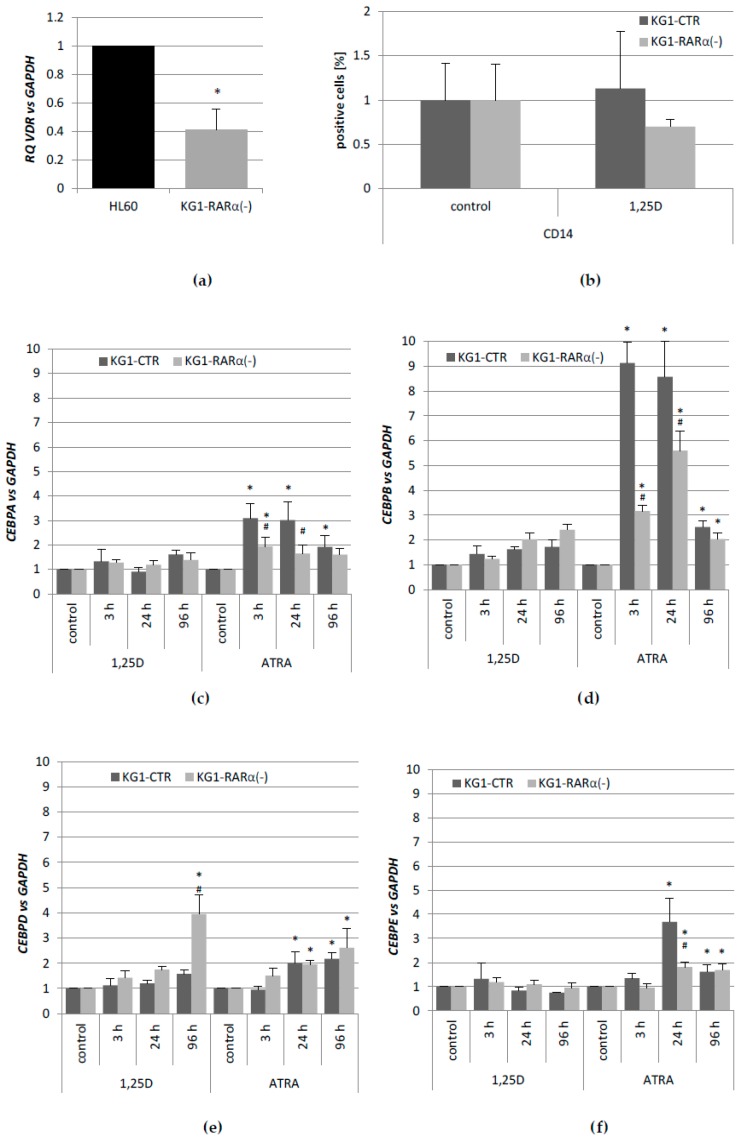
Responses to 1,25D and to ATRA in KG1 cells with silenced retinoic acid receptor α (*RARA*) gene. Expression of vitamin D receptor (*VDR*) gene in KG1-RARα(−) cells compared to HL60 cells, which were treated as calibrator (**a**). Differentiation of KG1 sublines in response to 1,25D. KG1-CTR and KG1-RARα(−) cells were stimulated with 10 nM 1,25D for 96 h and then the expression of CD14 differentiation marker was detected using flow cytometry (**b**). Expression of *CEBPA* (**c**), *CEBPB* (**d**), *CEBPD* (**e**), and *CEBPE* (**f**) genes in KG1-CTR and KG1-RARα(−). Cells were stimulated with 10 nM 1,25D or 1 µM ATRA and after desired time the expression of *CEBP* genes was measured by Real-time PCR. The bars represent mean values of the fold changes (±SEM) in mRNA levels relative to *GAPDH* mRNA levels. Expressions in control cells were treated as calibrators. Values that are significantly different from those obtained from respective controls cells are marked with asterisk (* *p* < 0.05); values that differ significantly from those obtained from respective KG1-CTR control cells are marked with hash (# *p* < 0.05).

In an attempt to elucidate whether the lack of one of the nuclear receptors VDR and RARα could be compensated by the other in terms of *CEBP* activation, we used KG1 sublines with silenced *RARA* gene (KG1-RARα(−)) and KG1 control cells (KG1-CTR), which were obtained before [31]. These cells have substantially reduced level of *RARA* gene expression and RARα protein, but also exhibit the increased expression of *VDR* gene and VDR protein, when compared to wild-type KG1 and KG1-CTR [31]. It should be noted that the expression of *VDR* gene in KG1-RARα(−) is still lower than in HL60 cells (Figure 3a), and it was not sufficient to induce antigen CD14 typical for monocytes (Figure 3b). The KG1 sublines were stimulated with 1,25D or ATRA in a similar manner as before. As presented in Figure 3e, KG1-RARα(−) cells started to be responsive to 1,25D, however in a manner that was different from HL60 cells. Only *CEBPD* gene became responsive to 1,25D in RARA silenced KG1 cells, and the expression of *CEBPB* remained at a control level. As expected, expression levels of *CEBPA*, *CEBPB*, and *CEBPE* were reduced in KG1-RARα(−) when compared to KG1-CTR cells after ATRA stimulation, especially at the early hours from stimulation (Figure 3c–f). Values of mRNA expression that were obtained using comparative quantification algorithm are presented in Table A3.

### 2.4. Effects of Silencing High VDR on Expression of CEBP Transcription Factor’s Genes in HL60 Cells

**Figure 4:**
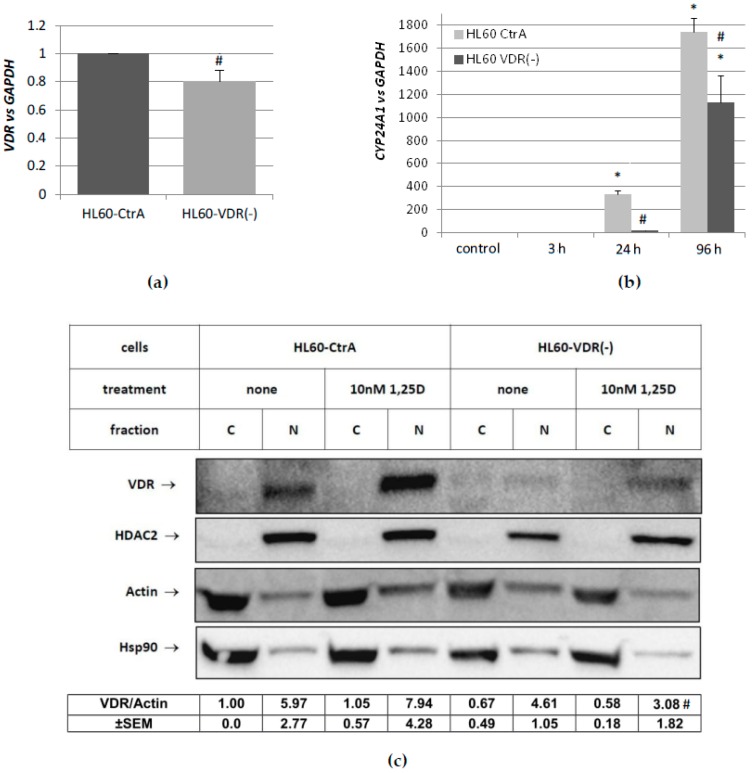
Generation of HL60 cells with reduced *VDR* expression. Constitutive expression of *VDR* gene in HL60-CtrA and HL60-VDR(−) cells. Expression in HL60-CtrA was treated as calibrator (**a**). Expression of *CYP24A1* was measured in both cell lines exposed to 10 nM 1,25D for different times (**b**). The levels of VDR protein were determined in the cytosol and nuclei of HL60-CtrA and HL60-VDR(−) cells by western blots after 10 nM 1,25D stimulation for 24h (c). Cell lysates were tested while using anti-VDR. Proper cell fractionation was revealed using anti-histone deacetylase 2 (anti-HDAC2), while proper lane loading using anti-Hsp90 and anti-actin. Values below the blots are means (±SEM), as obtained from five experiments. The bars represent mean values of the fold changes (±SEM) in mRNA levels relative to *GAPDH* mRNA levels. Values that are significantly different from those obtained from respective controls cells are marked with asterisk (* *p* < 0.05); values that differ significantly from those obtained from respective HL60-CtrA cells are marked with hash (# *p* < 0.05).

Having shown that KG1-RARα(−) cells demonstrate an altered *CEBP* expression profile, we decided to silence the expression of VDR gene in HL60 cells. The gene silencing was performed using shRNA plasmid and the scrambled shRNA plasmid, as described before [34]. This way, two HL60 sublines were generated: HL60-VDR(−) and HL60-CtrA. In order to validate whether the expression of *VDR* gene was indeed efficiently knocked down in HL60-VDR(−) cells, the mRNA and protein levels were compared to HL60-CtrA cells. Unfortunately, the silencing was far from complete and mRNA level was reduced to approximately 80% of the initial level (Figure 4a). In order to verify whether this reduction would lead to VDR-dependent effects, the expression of the gene that encodes 24-hydroxylase of 1,25D (CYP24A1) was tested in both HL60 sublines exposed to 10 nM 1,25D. CYP24A1 is the most strongly regulated out of all 1,25D-target genes and is the best measure of VDR’s activity [35]. 1,25D-induced expression of *CYP24A1* was significantly reduced in HL60-VDR(−) cells when compared to HL60-CtrA cells. Figure 4c shows that VDR protein content was also significantly reduced in the nuclei of HL60-VDR(−) cells to 77% in control cells, and to 39% after 1,25D treatment when compared to HL60-CtrA cells.

**Figure 5:**
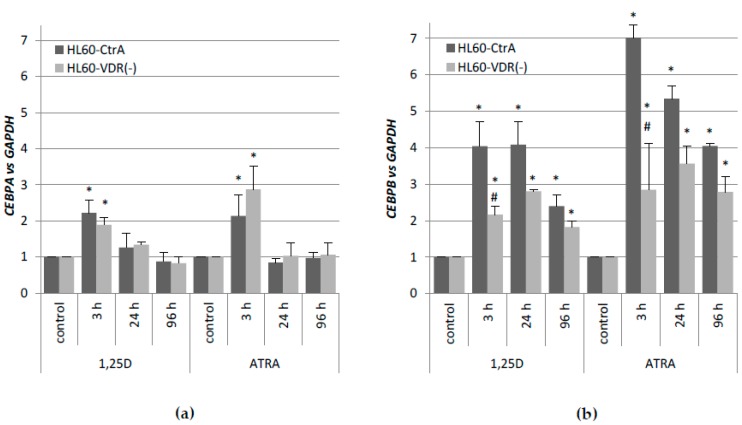
Expression of *CEBPA* (**a**) and *CEBPB* (**b**) genes in HL60-CtrA and HL60-VDR(−). Cells were exposed to 10 nM 1,25D or 1 µM ATRA and after desired time the expression of *CEBP* genes was measured by Real-time PCR. The bars represent mean values of the fold changes (±SEM) in mRNA levels relative to *GAPDH* mRNA levels. Expressions in control cells were treated as calibrators. Values that are significantly different from those obtained from respective controls cells are marked with an asterisk (* *p* < 0.05); values that differ significantly from those obtained from respective HL60-CtrA cells are marked with hash (# *p* < 0.05).

Even though HL60-VDR(−) subline was not entirely devoid of VDR, the cells were exposed 1 μM ATRA or 10 nM 1,25D in order to examine the expression of selected *CEBP* genes. *CEBPA* and *CEBPB* have been selected, since they are direct targets of VDR-dependent transcriptional regulation [36,37]. As presented in Figure 5a,b, the limited decrease of *VDR* expression level resulted in a reduced *CEBPA* and *CEBPB* expression levels in response to 1,25D when compared to HL60-CtrA cells. Interestingly, the response to ATRA in HL60-VDR(−) cells was different than in HL60-CtrA cells, and upregulated regarding *CEBPA* expression, while downregulated regarding *CEBPB* expression. Values of mRNA expression that were obtained using comparative quantification algorithm are presented in Table A4.

## 3. Discussion

The process of hematopoiesis leads to the acquisition of immune functions by terminally differentiated cells. Lineage selection within hematopoiesis depends on the appropriate levels of key transcription factors, which are regulated in response to numerous hematopoietic cytokines and interactions with bone marrow environment [38]. Transcription factors C/EBPα, C/EBPβ, and C/EBPδ have been demonstrated in granulocytes, monocytes, and eosinophils, as well as in myeloid progenitor cells [39]. C/EBPε has been identified as a critical regulator of terminal granulopoiesis [13]. Many genes that are important for myeloid functions contain in their promoters binding sites for C/EBP transcription factors [5]. In normal hematopoiesis, C/EBP transcription factors are produced in response to coordinated actions of cytokines and upstream transcription factors, and their activity is further modulated by posttranslational modifications [5].

C/EBPα seems to be the most important for normal blood development, since mutations in *CEBPA* gene lead to AML. *CEBPA* is mutated in around 13% of all AML patients [40], and mutations in this gene appear early, indicating at the driver role in leukemogenesis [41]. This is why the expression of *CEBPA* in AML patients has been extensively studied, and it has been shown to be downregulated by another driver of leukemogenesis, namely the AML-ETO fusion protein, which is present in 5–10% of patients with AML [42]. Later studies have documented that the downregulation of *CEBPA* also accompanies AML cases with inv(16), which creates *CBFB-MYH11* gene fusion and occurs in about 10% of AML patients [43]. All together, the above data show that more than 30% of patients with AML exhibit disturbances in expression of *CEBPA*. 

It has been shown that both 1,25D and ATRA are able to upregulate expression of C/EBP factors without the addition of hematopoietic cytokines [11,26,27,29]. Whether all the *CEBP* genes are direct targets for either RARα or VDR is not clear. Retinoic acid response elements (RAREs) have been found in the promoter of *CEBPE* gene, and not in other genes of this family [44], but at present, we know that RARα can bind to big variety of RAREs [45], which are sometimes located in a long distance from the transcription start [44]. *CEBPA* and *CEBPB* are direct targets of VDR-dependent transcriptional regulation [36,37], but it is not sure whether such a mechanism occurs also for *CEBPD*.

We thus wanted to find out how variable levels of VDR and RARα proteins affect the expression of *CEBP* genes, and whether the lack of one of the nuclear receptors that are mentioned above, could be compensated by the other. In the first place, we determined the expression profiles of these genes after stimulation with 1,25D or ATRA in HL60 and KG1 cells. These cells differ in basal levels of expression of VDR and RARα, and in susceptibility to 1,25D-induced differentiation. The basal levels of *VDR* and *RARA* mRNA expression in all of the cell lines that were used for the purpose of this research is presented in Table A5. HL60 cells, which have high level of VDR protein, respond to 1,25D with transient upregulation of *CEBPA*, and strong and sustained upregulation of *CEBPB*. It appeared that KG1 cells that have low level of VDR protein do not express *CEBP* genes in response to 1,25D at all. After the silencing of the *RARA* gene in KG1, these cells reduced the responsiveness to ATRA, but started to be responsive to 1,25D, most probably because of an increased expression of VDR [31]. The restored *VDR* expression level was not high enough to upregulate the expression of *CEBPB*, but it was sufficient to upregulate *CEBPD*. As presented here, the upregulation of *CEBPD* alone was not sufficient to complete the myeloid differentiation process. KG1 cells and HL60 cells are both responsive to ATRA, however, due to higher basal expression of *RARA*, KG1 cells respond stronger than HL60. In KG1 cells, the upregulation of *CEBPB* and *CEBPE* is approximately two times higher than in HL60 cells in response to ATRA; however, the kinetics of expression is similar.

Our results suggest that the ability of 1,25D or ATRA to effectively force the final myeloid differentiation of AML cells strongly depends on effective levels of nuclear receptors for these compounds. It also seems that expression of *CEBPB* is indispensable for the final effect of myeloid differentiation, and that VDR and RARα do not compensate each other in terms of the induction of *CEBP* expression. Our data are in agreement with the earlier findings that strong and sustained expression of *CEBPB*, when accompanied by transient expression of *CEBPA* leads to the differentiation towards monocytes [11], while, when accompanied by the sustained expression of *CEBPE*, it leads the differentiation process to granulocytes [46].

## 4. Materials and Methods

### 4.1. Cell Lines and Cultures

HL60 cells were from the local cell bank at the Institute of Immunology and Experimental Therapy in Wrocław, and KG1 cells were purchased from the German Resource Center for Biological Material (DSMZ GmbH, Braunschweig, Germany). The cells were grown in RPMI-1640 medium with 10% fetal bovine serum, 100 units/ml penicillin, and 100 µg/mL streptomycin (Sigma, St. Louis, MO, USA), and maintained at standard cell culture conditions.

### 4.2. Chemicals and Antibodies

1,25D was purchased from Cayman Europe (Tallinn, Estonia) and ATRA was from Sigma-Aldrich (St. Louis, MO, USA). The compounds were dissolved in an absolute ethanol to 1000× final concentrations, and subsequently, diluted in the culture medium to the required concentration.

### 4.3. cDNA Synthesis and Real-Time PCR

Total RNA was extracted from the cells treated with 1 µM ATRA or 10 nM 1,25D for different time points (3 h, 24 h, 96 h). Briefly, the isolation of total RNA, reverse transcription into cDNA, and Real-time PCR reactions were performed as published before [33], using CFX Real-time PCR System (Bio-Rad Laboratories Inc., Hercules, CA, USA). The sequences of *GAPDH*, *CYP24A1, VDR,* and *RARA* primers, and the reaction conditions were described previously [31,47]. The *CEBPA, CEBPB, CEBPD*, *CEBPE*, and *CEBPG* primers were obtained from RealTimePrimers.com (Real Time Primers, LLC, PA, USA). Their sequences are as follows: *CEBPA:* forward 5′-TTGGTGCGTCTAAGATGAGG-3′, reverse 5′-GGCAGGAAACCTCCAAATAA-3′; *CEBPB*: forward 5′-AACTCTCTGCTTCTCCCTCTG-3′, reverse 5′-AAGCCCGTAGGAACATCTTT-3′; *CEBPD*: forward 5′-ATCGACTTCAGCGCCTACAT-3′, reverse 5′-GCCTTGTGATTGCTGTTGAA-3′; *CEBPE*: forward 5′-GAGGAGGTTGCTCAGAGTGG-3′, reverse 5′-TCCTGGCCTATTCAGCAGTT-3′; *CEBPG*: forward 5′-GAACAACCCATTTTGCACTC-3′, reverse 5′-TGAAAGCCAGGAACAAAAAG-3′; *APDH*: forward 5′-CATGAGAAGTATGACAACAGCCT-3′, reverse 5′-AGTCCTTCCACGATACCAAAGT-3′. Quantification of gene expression was analyzed with either the ∆Cq (to present comparative quantification of expression levels) or with the ∆∆Cq (to present changes in expression induced by treatment) methods using *GAPDH* as the endogenous control Primers efficiencies were measured in all of the cell lines using Real-time PCR reaction based on the slope of the standard curve. The results were normalized to primer efficiencies to compare gene expression in different cell lines [48]. Real-time PCR assays were performed at least in triplicate.

### 4.4. Flow Cytometry

The expression of CD14 was determined by flow cytometry. The cells were incubated with 10 nM 1,25D for 96 h, then washed, and stained with 1 µL of Phycoerythrin labeled antibody (or the appropriate control immunoglobulins; both from ImmunoTools, Friesoythe, Germany) for 1 h on ice. Next, they were washed with ice-cold PBS supplemented with 0.1% BSA and suspended in 0.5 mL of PBS supplemented with 0.1% BSA prior to analysis on FACS Calibur flow cytometer (Becton–Dickinson, San Jose, CA, USA). Experiments were repeated at least three times. The acquisition parameters were set for an isotype control. Data analysis was performed with the use of WinMDI 2.8 software (freeware by Joseph Trotter).

### 4.5. Western Blotting

In order to obtain cytosolic and nuclear extracts, 5 × 10^6^ cells/sample were washed and lysed using NE-PER Nuclear and Cytoplasmic Extraction Reagents (Thermo Fisher Scientific Inc., Worcester, MA, USA), according to the user’s manual. Lysates were denatured by adding 5× sample buffer (1/4 volume of the lysate) and boiled for 5 min. 25 µL of each lysate were separated in SDS-PAGE and then electroblotted to PVDF membrane. The membranes were then dried and incubated sequentially with primary and a horseradish peroxidase-conjugated secondary antibody. The protein bands were visualized with a chemiluminescence. Then, the membranes were stripped, dried again, and probed with subsequent antibodies. Western blots were repeated five times.

### 4.6. Gene Silencing Reagents and Procedure

The *RARA* gene silencing in KG1 cells was described before [31]. The *VDR* gene silencing in HL60 cells was performed using shRNA plasmids and Neon^®^ Transfection System (Invitrogen™, Carlsbad, CA, USA) using control shRNA plasmid-A (sc-108060) and the *VDR* shRNA plasmid (sc-106692-SH; both from Santa Cruz Biotechnology, Inc., Dallas, TX, USA). The procedure of electrotransfection by Neon^®^ Transfection System was described before [49].

### 4.7. Statistical Analysis

For statistical analysis one-way ANOVA was used to test the null hypothesis that the samples in two or more groups are drawn from populations with the same mean values. When the ANOVA test had shown that the null hypothesis is not true, the Student’s *t*-test for independent samples was used to analyze the differences between the pairs of groups (Excel, Microsoft Office and free ANOVA Calculator: http://www.danielsoper.com/statcalc3/calc.aspx?id=43).

## Abbreviations

ATRA	all-*trans*-retinoic acid
1,25D	1α,25-dihydroxyvitamin D
C/EBP	CCAAT/enhancer-binding protein
RARs	retinoic acid receptors
AML	acute myeloid leukemia
HSCs	hematopoietic stem cells
APL	acute promyelocytic leukemia
VDR	vitamin D receptor
GAPDH	glyceraldehyde 3-phosphate dehydrogenase
SEM	standard error of the mean

## Appendix A

**Table A1:** Comparative quantification of *CEBP* genes related to a reference *GAPDH* gene in HL60 cells.

HL60		*CEBPA*	SEM	*CEBPB*	SEM	*CEBPD*	SEM	*CEBPE*	SEM	*CEBPG*	SEM
**control**		0.16	0.06	0.07	0.02	0.004	0.001	0.004	0.002	0.068	0.026
**1,25D**	**3 h**	0.33	0.07	0.19	0.03	0.003	0.001	0.003	0.002	0.085	0.051
	**24 h**	0.22	0.03	0.27	0.03	0.003	0.001	0.004	0.002	0.072	0.042
	**96 h**	0.15	0.05	0.18	0.02	0.003	0.001	0.004	0.001	0.046	0.030
**ATRA**	**3 h**	0.18	0.04	0.06	0.03	0.003	0.001	0.002	0.002	0.058	0.014
	**24 h**	0.25	0.07	0.30	0.14	0.002	0.000	0.007	0.008	0.061	0.020
	**96 h**	0.13	0.04	0.25	0.07	0.004	0.001	0.006	0.0071	0.055	0.012

**Table A2:** Comparative quantification of *CEBP* genes related to a reference *GAPDH* gene in KG1 cells.

KG1		*CEBPA*	SEM	*CEBPB*	SEM	*CEBPD*	SEM	*CEBPE*	SEM	*CEBPG*	SEM
**control**		0.025	0.016	0.039	0.000	0.0006	0.000	0.00003	0.0000	0.0037	0.0009
**1,25D**	**3 h**	0.035	0.022	0.060	0.000	0.0006	0.000	0.00003	0.0000	0.0041	0.0010
	**24 h**	0.032	0.015	0.063	0.000	0.0008	0.000	0.00003	0.0000	0.0031	0.0009
	**96 h**	0.031	0.058	0.053	0.000	0.0009	0.002	0.00002	0.0000	0.0034	0.0018
**ATRA**	**3 h**	0.026	0.011	0.039	0.000	0.0006	0.000	0.00002	0.0000	0.0023	0.0011
	**24 h**	0.063	0.030	0.496	0.000	0.0003	0.000	0.00009	0.0000	0.0023	0.0012
	**96 h**	0.050	0.027	0.284	0.000	0.0011	0.000	0.00013	0.0001	0.0031	0.0018

**Table A3:** Comparative quantification of *CEBP* genes related to a reference *GAPDH* gene in KG1-CTR and KG1-RARα(−) cells.

**KG1 CTR**		***CEBPA***	**SEM**	***CEBPB***	**SEM**	***CEBPD***	**SEM**	***CEBPE***	**SEM**
**control**		0.0061	0.0022	0.0199	0.0016	0.00002	0.0000	0.00003	0.0022
**1,25D**	**3 h**	0.0056	0.0019	0.0197	0.0013	0.00002	0.0000	0.00003	0.0018
	**24 h**	0.0048	0.0011	0.0282	0.0008	0.00002	0.0000	0.00002	0.0012
	**96 h**	0.0106	0.0017	0.0373	0.0012	0.00003	0.0000	0.00002	0.0016
**ATRA**	**3 h**	0.0153	0.0056	0.1484	0.0040	0.00001	0.0000	0.00003	0.0056
	**24 h**	0.0133	0.0080	0.1242	0.0056	0.00002	0.0000	0.00008	0.0079
	**96 h**	0.0134	0.0007	0.0577	0.0005	0.00003	0.0000	0.00005	0.0007
**KG1 RARα(−)**		***CEBPA***	SEM	***CEBPB***	SEM	***CEBPD***	SEM	***CEBPE***	SEM
**control**		0.0082	0.0023	0.0261	0.0016	0.00003	0.0000	0.00002	0.0022
**1,25D**	**3 h**	0.0123	0.0034	0.0377	0.0024	0.00003	0.0000	0.00003	0.0033
	**24 h**	0.0102	0.0034	0.0548	0.0024	0.00003	0.0000	0.00003	0.0033
	**96 h**	0.0120	0.0042	0.0660	0.0030	0.00007	0.0000	0.00002	0.0041
**ATRA**	**3 h**	0.0140	0.0038	0.0729	0.0027	0.00002	0.0000	0.00002	0.0037
	**24 h**	0.0135	0.0025	0.1457	0.0018	0.00003	0.0000	0.00004	0.0025
	**96 h**	0.0154	0.0036	0.0619	0.0025	0.00004	0.0000	0.00005	0.0034

**Table A4:** Comparative quantification of *CEBP* genes related to a reference *GAPDH* gene in HL60-CtrA and HL60-VDR(−) cells.

HL60 CtrA		*CEBPA*	SEM	*CEBPB*	SEM	HL60 VDR(−)		*CEBPA*	SEM	*CEBPB*	SEM
**control**		0.06228	0.0214	0.0753	0.0125	**control**		0.09500	0.0318	0.08958	0.0099
**1,25D**	**3 h**	0.05003	0.0089	0.3774	0.2312	**1,25D**	**3 h**	0.07767	0.0227	0.28449	0.0147
	**24 h**	0.07403	0.0172	0.2351	0.0788		**24 h**	0.12883	0.0455	0.41116	0.2031
	**96 h**	0.14063	0.0437	0.1749	0.0622		**96 h**	0.17163	0.0468	0.24263	0.0734
**ATRA**	**3 h**	0.06025	0.0180	0.1710	0.0118	**ATRA**	**3 h**	0.09056	0.0082	0.24728	0.0580
	**24 h**	0.05366	0.0167	0.2221	0.0188		**24 h**	0.09029	0.0166	0.40953	0.0871
	**96 h**	0.13521	0.0491	0.6720	0.0612		**96 h**	0.24610	0.0229	0.358334	0.2188

**Table A5:** Comparative quantification of *VDR* and *RARA* genes related to a reference *GAPDH* gene in all cell lines used in this study.

Cell Line	*VDR*	SEM	*RARA*	SEM
**HL60**	0.00048	0.00001	0.0679	0.001
**HL60 CtrA**	0.00045	0.00003	0.0600	0.003
**HL60 VDR(−)**	0.00035	0.00006	0.0638	0.003
**KG1**	0.00002	0.00001	0.1241	0.008
**KG1CTR**	0.00005	0.00000	0.1792	0.002
**KG1 RARα(−)**	0.00018	0.00003	0.0563	0.003
